# Orotic acid-treated hepatocellular carcinoma cells resist steatosis by modification of fatty acid metabolism

**DOI:** 10.1186/s12944-020-01243-5

**Published:** 2020-04-13

**Authors:** Johanna Matilainen, Anne-Mari Mustonen, Kirsi Rilla, Reijo Käkelä, Sanna P. Sihvo, Petteri Nieminen

**Affiliations:** 1 Faculty of Health Sciences, School of Medicine, Institute of Biomedicine, University of Eastern Finland, P.O. Box 1627, FI-70211 Kuopio, Finland; 2 Faculty of Science and Forestry, Department of Environmental and Biological Sciences, University of Eastern Finland, P.O. Box 111, FI-80101 Joensuu, Finland; 3 Faculty of Biological and Environmental Sciences, Molecular and Integrative Biosciences Research Programme, University of Helsinki, P.O. Box 65, FI-00014 Helsinki, Finland; 4 Helsinki Institute for Life Science (HiLIFE), Helsinki University Lipidomics Unit (HiLIPID), University of Helsinki, P.O. Box 65, FI-00014 Helsinki, Finland

**Keywords:** De novo lipogenesis, Hepatocyte, Inflammation, Lipidosis, Non-alcoholic fatty liver disease

## Abstract

**Background:**

Orotic acid (OA) has been intensively utilized to induce fatty liver in rats. Although the capacity of OA to cause steatosis is species-specific, previous in vitro studies indicate that humans could also be susceptible to OA-induced fatty liver. The aim of the present study was to re-elucidate the potential of OA exposure to modulate the cellular mechanisms involved in both non-alcoholic fatty liver disease pathogenesis and cellular protection from lipid accumulation. In addition, alterations in detailed fatty acid (FA) profiles of cells and culture media were analyzed to assess the significance of lipid metabolism in these phenomena.

**Methods:**

In our experiments, human hepatocellular carcinoma HepG2 cells were exposed to OA. Bacterial endotoxin, lipopolysaccharide (LPS), was used to mimic hepatic inflammation. The lipogenic and inflammatory effects of OA and/or LPS on cells were assessed by labeling cellular lipids with Nile red stain and by performing image quantifications. The expression levels of key enzymes involved in de novo lipogenesis (DNL) and of inflammatory markers related to the disease development were studied by qRT-PCR. FA profiles of cells and culture media were determined from total lipids with gas chromatography–mass spectrometry.

**Results:**

Our data indicate that although OA possibly promotes the first stage of DNL, it does not cause a definite lipogenic transformation in HepG2 cells. Reduced proportions of 16:0, increased stearoyl-Coenzyme A desaturase 1 mRNA expression and relatively high proportions of 16:1n-7 suggest that active delta9-desaturation may limit lipogenesis and the accumulation of toxic 16:0. Inflammatory signaling could be reduced by the increased production of long-chain n-3 polyunsaturated FA (PUFA) and the active incorporation of certain FA, including 18:1n-9, into cells. In addition, increased proportions of 20:4n-6 and 22:6n-3, total PUFA and dimethyl acetal 18:0 suggest that OA exposure may cause increased secretion of lipoproteins and extracellular vesicles.

**Conclusions:**

The present data suggest that, apart from the transcription-level events reported by previous studies, modifications of FA metabolism may also be involved in the prevention of OA-mediated steatosis. Increased delta9-desaturation and secretion of lipoproteins and extracellular vesicles could offer potential mechanisms for further studies to unravel how OA-treated cells alleviate lipidosis.

## Background

Non-alcoholic fatty liver disease (NAFLD), a condition characterized by excess hepatic lipid accumulation in the absence of significant alcohol consumption, is one of the most common causes of chronic liver disease worldwide [1]. The global prevalence of NAFLD has been estimated to be 25% among adults, with the highest rates in the Middle East (32%) and South America (31%). Obesity, metabolic syndrome, type 2 diabetes mellitus and hypertension are the most important risk factors for NAFLD [2]. The accumulation of fat in hepatocytes can arise from several sources, including dietary fat, mobilization of adipose tissue triacylglycerols (TAG) and de novo lipogenesis (DNL), the synthesis of fatty acids (FA) from acetyl-coenzyme A (acetyl-CoA) [3]. During DNL, acetyl-CoA subunits are first converted to malonyl-CoA by acetyl-CoA carboxylase, followed by the generation of 16:0 by fatty acid synthase (FASN) [4]. In addition, decreased *β*-oxidation in mitochondria and TAG export via very-low-density lipoprotein (VLDL) particles are associated with the disease. Accumulated FA are subsequently esterified into TAG and stored as lipid droplets inside hepatocytes [5].

While TAG are the most abundant lipid components in steatosis droplets, there is growing evidence that their accumulation may represent a protective mechanism against cell injury and disease progression to non-alcoholic steatohepatitis (NASH). Lipid droplet TAG provide safe buffering capacity, which limits the cellular pools of toxic non-TAG lipids and free FA and, thus, protects cells against lipotoxic effects [6]. The activation of several cellular stress pathways by toxic lipids is followed by organelle dysfunction, cellular injury and, ultimately, cell death [7]. The lipids that can have toxic effects include free saturated FA (SFA), diacylglycerols, free cholesterol, ceramides and sphingolipids. In line with this, NAFLD is characterized with increased levels of SFA, such as 16:0, in TAG and diacylglycerol fractions of the liver, and these lipids have been demonstrated to induce endoplasmic reticulum (ER) stress, apoptosis and c-Jun NH_2_-terminal kinase activation in hepatocytes [8–10]. On the other hand, long-chain n-3 polyunsaturated FA (PUFA) seem to alleviate the effects caused by the toxic lipid accumulation. Indeed, characteristic features of NAFLD include a depletion of n-3 PUFA and an increased n-6/n-3 PUFA ratio in the liver [10, 11], suggesting that reduced n-3 PUFA levels play a role in NAFLD pathogenesis and progression. For instance, 20:5n-3 decreases the production of inflammatory mediators by adipocytes, which could alleviate the inflammation of adipose tissue and the subsequent fat deposition in the liver [12]. Furthermore, n-3 PUFA reduce the activation of sterol regulatory element binding protein-1c (SREBP-1c) mediating the activity of lipogenic enzymes, e.g., acetyl-CoA carboxylase and FASN in the liver [13, 14]. In addition, they can activate peroxisome proliferator-activated receptor (PPAR)-*α*, which induces mitochondrial *β*-oxidation [13]. Moreover, n-3 PUFA-derived lipid mediators, e.g., protectins, resolvins and maresins, alleviate DNA damage and oxidative stress in hepatocytes, as well as inflammation mediated by Kupffer cells [15]. Conversely, n-6 PUFA-derived lipid mediators generally possess pro-inflammatory and pro-thrombotic activities [16] albeit, e.g., lipoxins can have pro-resolving properties [15].

Orotic acid (OA), an intermediate in the synthesis of pyrimidine nucleotides, has been intensively utilized to induce fatty liver in rats [17–19]. FASN activity, TAG accumulation, decreased mitochondrial *β*-oxidation and decreased secretion of VLDL and low-density lipoproteins (LDL) were identified as the main events promoting fatty liver due to OA exposure. However, OA has failed to induce fatty liver in other rodents, chicken, rabbits, pigs or monkeys, indicating that the OA-mediated hepatic steatosis could be restricted to one or a few species. In vivo experiments with rats reveal that the mechanisms by which OA-supplemented diet induces fatty liver include enhanced DNL via the activation of SREBP-1c [20]. The OA-induced SREBP-1c activity occurs via serine/threonine kinase 11 degradation, AMP-activated protein kinase (AMPK) inhibition and the subsequent mammalian target of rapamycin (mTOR) activation [21]. The authors suggested that the effects of OA in rats arise from the regulation of AMPK and SREBP-1c, as these were not affected by OA in mouse primary hepatocytes. As OA also inhibited AMPK and activated mTOR in human hepatocellular carcinoma (HCC) cells, as found for rat hepatocytes, the authors stated that humans could also be susceptible to OA-induced fatty liver. Despite this, previous research evaluating OA-mediated hepatocyte lipidosis in cell types other than hepatocytes of rat origin remains sparse. In addition, to our best knowledge, there are no previous data about the detailed FA composition of the cells nor about how the available FA are processed in these cells.

We re-evaluated the potential of OA exposure as a model for early-stage hepatocyte steatosis and its prevention and tested if it is useful for investigating the mechanisms involved in both NAFLD pathogenesis and cellular protection. For this goal, we used the human HCC HepG2 cell line, in which lipogenic events were initiated by OA. Bacterial endotoxin, lipopolysaccharide (LPS), was used to study the potential combinatory effects of OA and an inflammatory agent, as inflammation is involved in further disease progression to NASH and fibrosis [22]. In addition to investigating changes at the transcriptional level, alterations in detailed FA profiles of cells and culture media were studied. Based on previous reports, it was hypothesized that, in addition to protective events at the transcriptional level of DNL, modifications of FA metabolism may also be involved in the process of resisting lipid accumulation in HepG2 cells.

## Methods

### Cell culture and OA and LPS treatments

Human HepG2 cells (ECACC 85011430) were purchased from Sigma-Aldrich (St. Louis, MO, USA) and cultured in Eagle’s Minimum Essential Medium (EMEM) without L-Glutamine (Lonza, Verviers, Belgium) supplemented with 10% fetal bovine serum (HyClone, Logan, UT, USA), 2 mM glutamine (EuroClone, Pero, Italy), 0.1 mM Non-Essential Amino Acids NEAA (Lonza), 100 U/ml penicillin and 100 μg/ml streptomycin (EuroClone). The cells were incubated at 37 °C in 5% CO_2_. For the treatments, HepG2 cells were transferred into 12-well plates (1.0 × 10^5^ cells per well) and incubated for overnight. The cells were subsequently treated with 500 μM OA (O2750, Sigma-Aldrich), 50 ng/ml LPS (L5543, Sigma-Aldrich) or with these both for 5 days in the culture medium. OA was dissolved in DMSO, from which the working stock was prepared by diluting it in cell culture medium. Solvent controls were included in the treatments. After 48-h incubation, the culture medium was replaced with newly-prepared medium and the incubation proceeded for 5 days.

### Nile red and propidium iodide (PI) staining of cells and image quantifications

For Nile red and PI staining, the cells treated with OA and/or LPS as stated above were plated on chamber slides (Ibidi GmbH, Martinsried, Germany). For Nile red staining, the cells were then fixed with 4% paraformaldehyde for 20 min at RT. After phosphate buffered saline (PBS) washes, the intracellular lipids were labelled with 7.5 μM Nile red stain (72485, Sigma-Aldrich) for 30 min, and cell nuclei were stained with DAPI (1 μg/ml, Sigma-Aldrich). For PI staining that demonstrates cell viability, nuclei of living cells were first stained with NucBlue® Live Cell Stain ReadyProbes™ reagent (R37605, Life Technologies, Eugene, OR, USA) at RT for 5 min. Cells were then stained with 60 μM PI (SC-3541, Santa Cruz Biotechnology, Santa Cruz, CA, USA) at 37 °C for 5 min and washed with PBS. Cells were visualized with 40× NA 1.3 objective on a Zeiss Axio Observer inverted microscope equipped with a Zeiss LSM 800 confocal module (Carl Zeiss Microimaging GmbH, Jena, Germany). The quantitative analyses of the Nile red signal were performed with the ZEN 2009 software (Carl Zeiss Microimaging GmbH). The threshold values for the fluorescence signal were optimized to separate the signal from the background. To obtain total intensities of Nile red stain, the number of signal particles was multiplied by the mean intensity of the fluorescence signal. Data were expressed as mean intensity % of control measurements. PI-positive cells were manually counted from confocal images, and data were expressed as % PI-positive cells from counted cells.

### TAG level measurement

Total TAG levels in cells were analyzed by colorimetric assay (10010303, Cayman Chemical, Ann Arbor, MI, USA). For the OA treatments, HepG2 cells were transferred into 15-cm culture dishes (2.4 × 10^6^ cells per dish) and treated with 500 μM OA for 5 days as described above. For each sample, 14.4 × 10^6^ cells were plated. Cells were collected and lysates prepared according to assay protocol, and stored at − 70 °C for the subsequent TAG level measurements. During sample collections, cells were counted for the subsequent data normalizations. Data were expressed as the amount of TAG (mg) per 1 × 10^6^ cells.

### Gas chromatography–mass spectrometry

After the OA and/or LPS treatments, 2 ml of culture medium was taken for the subsequent gas chromatography (GC) FA analysis. The FA composition was also analyzed from the raw medium. Cells were washed with PBS, detached with trypsin and centrifuged (5000 rpm × 5 min). After centrifugation, cell pellets were stored at − 70 °C until the FA determinations. For the analyses, excess water was first removed from the subsamples of cells and media by nitrogen stream followed by transmethylation in methanolic H_2_SO_4_ under nitrogen atmosphere [23]. The formed FA methyl esters (FAME) were extracted with hexane and analyzed by a Shimadzu GC-2010 Plus gas chromatograph (Shimadzu, Kyoto, Japan) with a flame ionization detector (FID). The transmethylation of alkenyl chains from plasmalogen phospholipids produces dimethyl acetals (DMA), which were also included in the analysis. The FAME and DMA structures were confirmed by using electron impact mass spectra recorded by a Shimadzu GCMS-QP2010 Ultra with a mass selective detector (MSD). The GC-FID and GC-MSD systems were equipped with ZB-wax capillary columns (Phenomenex, Torrance, CA, USA). The resulting chromatographic peaks were manually integrated with the GCsolution software (*v*2.41.00) by Shimadzu. The results are expressed as mol-% in total lipid side chains of the cells and media. The n-6 and n-3 PUFA product/precursor ratios were calculated as follows: 20:4n-6/18:2n-6 and (20:5n-3 + 22:6n-3)/18:3n-3.

### qRT-PCR

OA and/or LPS-treated cells seeded on 12-well plates were lysed with TRI Reagent® (Molecular Research Center, Cincinnati, OH, USA) for mRNA expression analyses. Total RNA was extracted with chloroform–isopropyl alcohol according to standard procedures, washed with 75% ethanol, dissolved in nuclease-free water and stored at − 70 °C. For cDNA synthesis, 1 μg of total RNA was synthesized using the Verso™ SYBR Green Master kit (Thermo Fisher Scientific, Vilnius, Lithuania) according to the manufacturer’s protocol. The qRT-PCR was performed with Fast Start Universal SYBR Green mix reagent and LightCycler 480 PCR system (Roche Applied Science, Indianapolis, IN, USA). Total fold changes were calculated using the 2(−ΔΔCt) method, where ΔΔCt is the Ct (treatment) – Ct (control), ΔCt is Ct (target gene) – Ct (reference gene), and Ct is the number of PCR cycles needed to cross the detection threshold. Ribosomal protein, Large, P0 (Rplp0) was used as the reference gene. The primer sequences are given in Table 1. The data were expressed as fold changes compared to control.

**Table 1:** Quantitative real-time PCR primer sequences

Gene		Primer sequences	
Acetyl-CoA carboxylase *α*	ACACA	Forward 5′	TGCTCGTGGATGAACCAGAC
		Reverse 5′	TCCAAAAAGACCTAGCCCTCAA
Acetyl-CoA carboxylase *β*	ACACB	Forward 5′	ACTCTGTTGCTGGCTCATCT
		Reverse 5′	GACATGCTCGGCCTCATAGT
Fatty acid synthase	FASN	Forward 5′	CAGGAGTTCTGGGACAACCT
		Reverse 5′	CCTCGGAGTGAATCTGGGTT
Interleukin 6	IL-6	Forward 5′	TGCAATAACCACCCCTGACC
		Reverse 5′	GTGCCCATGCTACATTTGCC
Interleukin 8	IL-8	Forward 5′	CAGTGGACCACACTGCGCCAA
		Reverse 5′	TCCACAACCCTCTGCACCCAGTT
Stearoyl-Coenzyme A desaturase	SCD1	Forward 5′	GGGTGTGCTGACAACTTAGC
		Reverse 5′	TAGGGTCTCAGGTGCAAAGG
Ribosomal protein, Large, P0	Rplp0	Forward 5′	AGATGCAGCAGATCCGCAT
		Reverse 5′	GTGGTGATACCTAAAGCCTG

More detailed gene expression analyses were performed by using Qiagen RT^2^ Profiler™ Human Fatty Liver (330231 PAHS-157ZA, Qiagen, Hilden, Germany) PCR Arrays. Each array plate comprised of primers specific to 84 genes involved in the mechanisms of NAFLD and hepatic insulin resistance, housekeeping genes and appropriate controls. Expression difference was calculated as fold-difference using the ΔΔCt method, where data were normalized to the average Ct value of five housekeeping genes. The data were expressed as mean fold changes compared to control.

### Statistical testing

Comparisons between the treatments were performed with the Kruskal–Wallis nonparametric analysis of variance (ANOVA; IBM SPSS *v*21.0 software, IBM, Armonk, NY, USA). Nonparametric tests between two study groups were performed with the Mann–Whitney U test. The *p* value < 0.05 was considered statistically significant. The results are presented as the mean ± SEM. To perform a general assessment of the FA and DMA profiles, we also performed the discriminant analysis by classifying the composition data by discriminant functions to see how the cell and medium samples in the different treatment groups differed from one another, which variables separated them most clearly and how well the analysis was able to classify the samples into their respective treatments: control, OA, LPS or OA–LPS.

## Results

### Lipogenic and inflammatory responses in OA and/or LPS-treated HepG2 cells

**Fig. 1:**
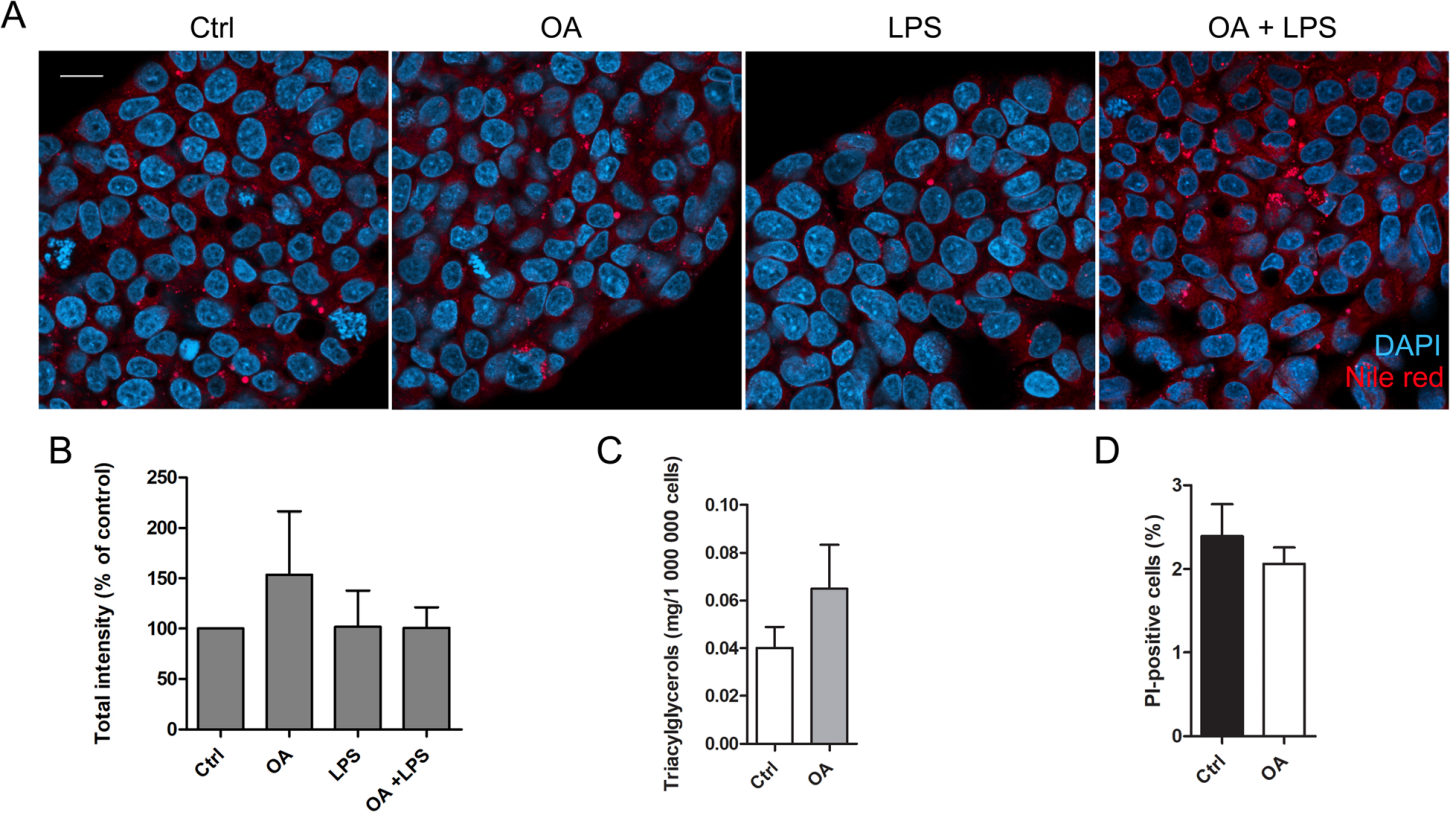
Nile red staining, triacylglycerol (TAG) measurements and propidium iodide (PI) staining. HepG2 cells were treated with either orotic acid (OA; 500 μM), lipopolysaccharide (LPS; 50 ng/ml) or both, and fixed after 5 days. Cellular lipids were stained with Nile red stain, and cells were observed with a fluorescence confocal microscope (**a**). Scale bar 20 μm, DAPI = nuclei. The total intensity (number of pixels × mean intensity) of Nile red stain signal was quantified with the ZEN 2009 software (**b**). Quantification results were normalized by dividing the treatment sample values by control measurements. Results are presented as mean % of control measurements + SEM and counted from five independent experiments, each including at least 10 images per sample. Total TAG levels in OA-treated cells were measured by colorimetric assay (**c**). The amount of TAG (mg) was normalized to cell count. Results are presented as mean + SEM and counted from three independent experiments. Viability of OA-treated cells was evaluated by PI staining (**d**). OA-treated cells were stained with PI stain and observed with a fluorescence confocal microscope. PI-positive cells were manually counted from three independent experiments, each including at least 1300 cells per sample. Results are presented as mean % of PI-positive cells from total cell count + SEM

The ability of our OA–LPS model to induce lipidosis in HepG2 cells was evaluated by fluorescent labelling of intracellular lipids by Nile red stain followed by confocal microscopy, visual observation and image quantifications (Fig. 1a–b). Even after 5 days of incubation with OA and LPS, there was no significant fat accumulation. While the treatment with OA alone tended to induce slightly increased levels of intracellular lipids, it did not reach statistical significance. Moreover, LPS alone did not induce or aggravate lipidosis. The measurement of total TAG levels by colorimetric assay further confirmed that OA did not increase the amount of lipids (Fig. 1c; Mann–Whitney U test, *p* = 0.827). PI-staining revealed that OA did not affect cell viability (Fig. 1d).

**Fig. 2:**
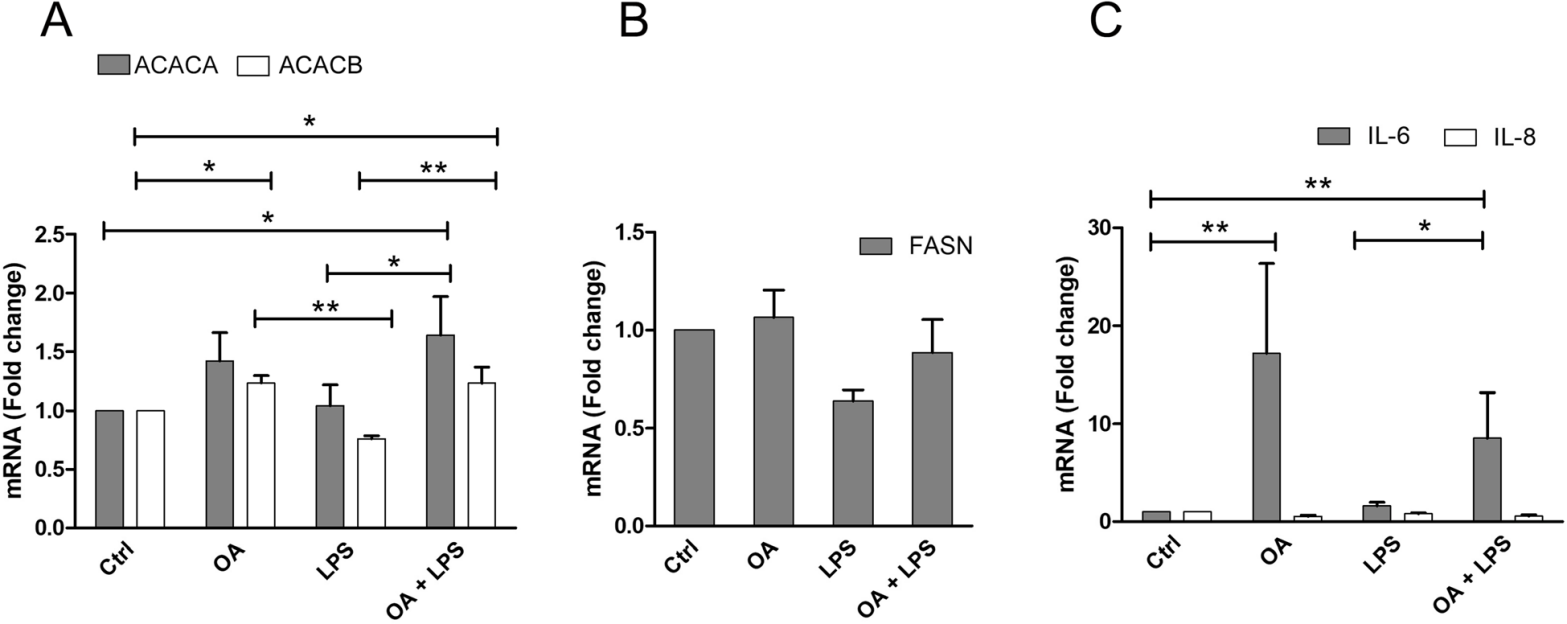
The mRNA expression levels of ACACA, ACACB, FASN, IL-6 and -8. The mRNA expression levels of **a** acetyl-coenzyme A carboxylase *α* (ACACA) and *β* (ACACB), **b** fatty acid synthase (FASN) and **c** interleukin (IL) -6 and -8 in HepG2 cells treated with OA (orotic acid; 500 μM), LPS (lipopolysaccharide; 50 ng/ml) or both. The means + SEM of six independent experiments are shown. *p < 0.05, **p = 0.001–0.01

OA and OA–LPS significantly increased the expression of acetyl-CoA carboxylase *α* (ACACA) and *β* (ACACB) (Fig. 2a). However, despite the elevated levels of these enzymes responsible for the first committed step of FA synthesis, the expression of FASN was not affected in OA or in OA–LPS-treated cells (Fig. 2b). Moreover, OA or OA–LPS treatments did not induce any changes in the activity of FASN assessed by activity-based protein profiling (data not shown). In addition, the mRNA levels of interleukin (IL)-6, but not those of IL-8, increased in cells treated with OA alone and together with LPS (Fig. 2c).

#### Increased Δ9-desaturation mRNA expression in HepG2 cells

**Fig. 3:**
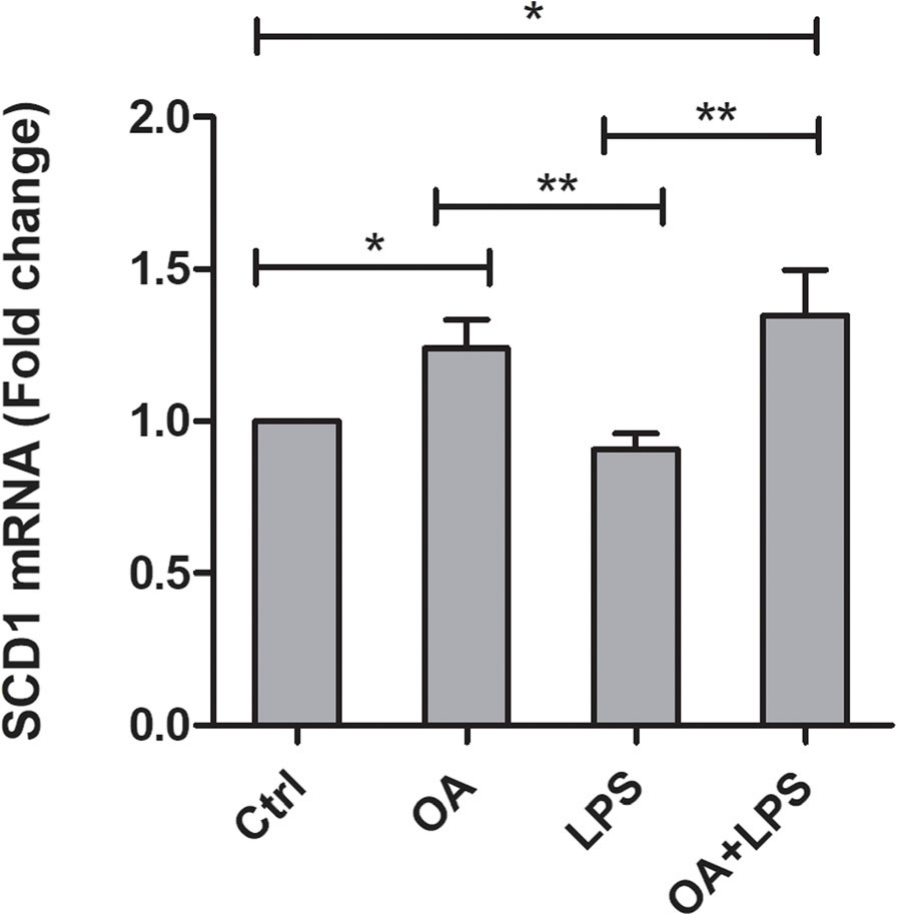
The mRNA expression levels of SCD1. The mRNA expression levels of stearoyl-Coenzyme A desaturase 1 (SCD1) in HepG2 cells treated with OA (orotic acid; 500 μM), LPS (lipopolysaccharide; 50 ng/ml) or both. The means + SEM of six independent experiments are shown. *p < 0.05, **p = 0.001–0.01

Slightly increased proportions of 16:1n-7 together with significantly reduced levels of 16:0 in OA- and OA–LPS-treated cells led us to further examine whether Δ9-desaturation could be promoted by OA. We studied the mRNA expression of stearoyl-Coenzyme A desaturase 1 (SCD1; Fig. 3). The expression of SCD1 was significantly higher in OA- and OA–LPS-treated cells when compared to control cells, while the expression in LPS-treated cells remained similar to the expression in control cells.

In order to further evaluate the effects of OA on targets relevant to fatty liver, RT^2^ Profiler™ PCR Array Human Fatty Liver analyses were performed for control and OA-treated cells. According to these analyses, OA did not cause any statistically significant changes in gene expression, except of the 0.5-fold change of protein tyrosine phosphatase, non-receptor type 1 (Mann–Whitney U test, *p* = 0.046) (Additional file 1).

#### Lipid manifestations of OA and/or LPS treatments

**Fig. 4:**
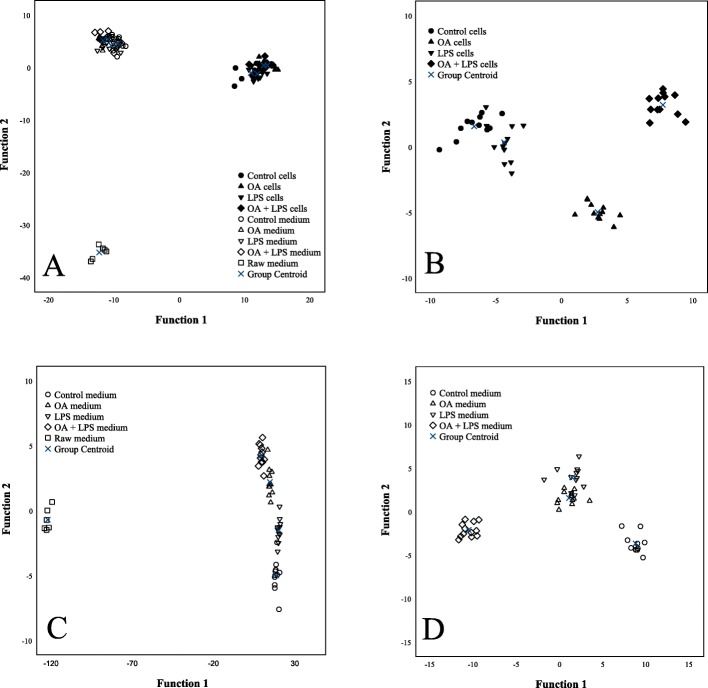
Discriminant analyses of FA results. Discriminant analyses depicting the classification of fatty acid signatures of HepG2 cells and culture media in the different treatment groups based on discriminant functions 1 and 2. **a** represents all samples, **b** represents HepG2 cells and **c** all media, while in **d** raw medium is excluded. Note that the scaling varies between panels in the x- and y-axes. With the first two functions, 97.1% of the variance was explained in panel **a**, 95.2% in panel **b**, 99.6% in panel **c** and 91.1% in panel **d**. Black symbols = HepG2 cells, white symbols = culture media

The results of the discriminant analyses of the FA signatures are visualized in Fig 4a–d. Control and LPS-treated HepG2 cells were grouped together whereas OA- and OA–LPS-treated cells were separated from each other and from control and LPS-treated cells (Fig. 4b). The individual FA with the largest separation power included 20:4n-6, 20:3n-6, 22:6n-3, 24:1n-9 and 16:1n-9. The analysis classified 95.7% of the samples correctly into their respective treatment groups. Compared to control cells, OA- and OA–LPS-treated cells had higher proportions of DMA 18:0, individual FA 20:3n-6, 20:4n-6, 20:5n-3 and 22:6n-3, the FA structural categories n-3 PUFA, n-6 PUFA and total PUFA, as well as higher product/precursor ratios of n-6 PUFA (Table 2). In contrast, they showed lower percentages of 16:0, 18:1n-5 and total SFA and lower product/precursor ratios of n-3 PUFA. In addition, OA-treated cells had higher unsaturated FA (UFA)/SFA ratios and n-3/n-6 PUFA ratios compared to control cells, and the percentage of 24:1n-9 was higher in OA–LPS-treated cells than in control cells. The proportion of 22:1n-9 was lower in LPS-treated cells compared to control cells.

**Table 2:** Fatty acyl and alkenyl chain profiles (mol-%) in HepG2 cells

	Control cells	OA cells	LPS cells	OA–LPS cells	*p*
14:0	2.676 ± 0.056	2.921 ± 0.267	2.785 ± 0.125	2.634 ± 0.133	0.084
14:1n-5	0.268 ± 0.039	0.528 ± 0.246	0.305 ± 0.106	0.289 ± 0.033	0.443
15:0	0.388 ± 0.033	0.435 ± 0.114	0.358 ± 0.034	0.350 ± 0.029	0.815
DMA 16:0	0.049 ± 0.010	0.075 ± 0.026	0.060 ± 0.023	0.058 ± 0.009	0.662
**16:0**	**27.653 ± 0.513**^**C**^	**24.972 ± 0.439**^**A**^	**27.159 ± 0.428**^**BC**^	**25.583 ± 0.336**^**AB**^	**< 0.001**
16:1n-9	2.163 ± 0.086	2.107 ± 0.082	2.138 ± 0.027	2.069 ± 0.070	0.755
16:1n-7	8.860 ± 0.532	10.466 ± 0.600	9.357 ± 0.568	9.960 ± 0.547	0.434
16:1n-5	0.559 ± 0.030	0.670 ± 0.119	0.561 ± 0.029	0.543 ± 0.033	0.911
17:0*i*	0.101 ± 0.011	0.195 ± 0.078	0.127 ± 0.017	0.105 ± 0.014	0.627
17:0*ai*	0.228 ± 0.026	0.281 ± 0.079	0.208 ± 0.027	0.208 ± 0.015	0.751
17:0	0.261 ± 0.010	0.262 ± 0.053	0.260 ± 0.033	0.387 ± 0.113	0.133
17:1n-8	0.585 ± 0.038	0.601 ± 0.024	0.565 ± 0.030	0.601 ± 0.037	0.878
**DMA 18:0**	**0.048 ± 0.003**^**A**^	**0.106 ± 0.030**^**B**^	**0.067 ± 0.020**^**A**^	**0.080 ± 0.011**^**B**^	**0.003**
DMA 18:1n-9	0.018 ± 0.003	0.071 ± 0.041	0.026 ± 0.005	0.034 ± 0.007	0.090
DMA 18:1n-7	0.014 ± 0.002	0.035 ± 0.013	0.018 ± 0.003	0.020 ± 0.003	0.257
18:0	7.091 ± 0.456	6.527 ± 0.336	6.646 ± 0.432	6.981 ± 0.382	0.919
18:1n-9	20.164 ± 0.358	19.320 ± 0.563	20.176 ± 0.174	19.699 ± 0.205	0.307
18:1n-7	15.077 ± 0.293	14.099 ± 0.510	15.344 ± 0.162	14.545 ± 0.361	0.326
**18:1n-5**	**0.846 ± 0.020**^**B**^	**0.795 ± 0.059**^**A**^	**0.895 ± 0.017**^**B**^	**0.751 ± 0.033**^**A**^	**0.003**
18:2n-7	0.089 ± 0.008	0.114 ± 0.029	0.089 ± 0.007	0.069 ± 0.006	0.124
18:2n-6	1.162 ± 0.038	1.164 ± 0.088	1.209 ± 0.035	1.052 ± 0.066	0.255
18:3n-6	0.066 ± 0.010	0.138 ± 0.037	0.100 ± 0.016	0.100 ± 0.023	0.371
19:1n-8	0.058 ± 0.007	0.067 ± 0.009	0.051 ± 0.005	0.071 ± 0.004	0.085
**18:3n-3**	**0.194 ± 0.021**^**AB**^	**0.224 ± 0.020**^**AB**^	**0.185 ± 0.019**^**A**^	**0.222 ± 0.011**^**B**^	**0.042**
18:2*c*9*t*11	0.054 ± 0.016	0.104 ± 0.040	0.054 ± 0.011	0.070 ± 0.017	0.684
20:0	0.239 ± 0.014	0.385 ± 0.139	0.220 ± 0.010	0.242 ± 0.014	0.298
20:1n-11	0.279 ± 0.091	0.278 ± 0.083	0.177 ± 0.068	0.275 ± 0.085	0.866
20:1n-9	0.625 ± 0.038	0.626 ± 0.042	0.652 ± 0.041	0.589 ± 0.031	0.697
20:1n-7	0.424 ± 0.052	0.428 ± 0.056	0.430 ± 0.048	0.403 ± 0.049	0.927
20:2n-9	0.173 ± 0.026	0.200 ± 0.037	0.215 ± 0.033	0.152 ± 0.021	0.461
20:2n-6	2.081 ± 0.079	1.838 ± 0.187	2.088 ± 0.127	1.993 ± 0.179	0.466
20:3n-9	0.243 ± 0.015	0.293 ± 0.075	0.259 ± 0.020	0.244 ± 0.020	0.514
**20:3n-6**	**0.617 ± 0.022**^**A**^	**0.846 ± 0.055**^**B**^	**0.645 ± 0.023**^**A**^	**0.815 ± 0.047**^**B**^	**< 0.001**
**20:4n-6**	**2.632 ± 0.107**^**A**^	**3.617 ± 0.251**^**B**^	**2.658 ± 0.140**^**A**^	**3.682 ± 0.251**^**B**^	**< 0.001**
20:4n-3	0.049 ± 0.007	0.071 ± 0.014	0.053 ± 0.008	0.059 ± 0.008	0.384
**20:5n-3**	**0.254 ± 0.018**^**A**^	**0.426 ± 0.028**^**B**^	**0.263 ± 0.014**^**A**^	**0.365 ± 0.032**^**B**^	**< 0.001**
22:0	0.228 ± 0.011	0.289 ± 0.066	0.205 ± 0.018	0.234 ± 0.012	0.343
22:1n-11	0.192 ± 0.036	0.169 ± 0.029	0.114 ± 0.016	0.206 ± 0.052	0.206
**22:1n-9**	**0.016 ± 0.002**^**B**^	**0.024 ± 0.011**^**B**^	**0.009 ± 0.001**^**A**^	**0.020 ± 0.003**^**B**^	**0.011**
22:1n-7	0.078 ± 0.011	0.132 ± 0.035	0.121 ± 0.050	0.131 ± 0.053	0.258
22:4n-6	0.037 ± 0.006	0.064 ± 0.018	0.037 ± 0.006	0.052 ± 0.011	0.714
22:5n-3	0.249 ± 0.012	0.339 ± 0.031	0.254 ± 0.014	0.284 ± 0.022	0.120
**24:0**	**0.209 ± 0.029**^**AB**^	**0.210 ± 0.023**^**B**^	**0.161 ± 0.018**^**A**^	**0.219 ± 0.015**^**B**^	**0.047**
**22:6n-3**	**2.203 ± 0.106**^**A**^	**2.913 ± 0.190**^**B**^	**2.178 ± 0.114**^**A**^	**2.900 ± 0.208**^**B**^	**< 0.001**
**24:1n-9**	**0.504 ± 0.037**^**A**^	**0.577 ± 0.045**^**AB**^	**0.524 ± 0.119**^**A**^	**0.654 ± 0.059**^**B**^	**0.009**
**SFA**	**39.073 ± 0.838**^**B**^	**36.478 ± 0.408**^**A**^	**38.128 ± 0.672**^**AB**^	**36.943 ± 0.494**^**A**^	**0.032**
MUFA	50.696 ± 0.726	50.886 ± 0.550	51.416 ± 0.635	50.805 ± 0.712	0.910
**PUFA**	**10.103 ± 0.236**^**A**^	**12.350 ± 0.271**^**B**^	**10.286 ± 0.345**^**A**^	**12.060 ± 0.565**^**B**^	**< 0.001**
**n-6 PUFA**	**6.595 ± 0.140**^**A**^	**7.665 ± 0.188**^**B**^	**6.736 ± 0.235**^**A**^	**7.695 ± 0.335**^**B**^	**0.001**
**n-3 PUFA**	**2.949 ± 0.126**^**A**^	**3.973 ± 0.202**^**B**^	**2.933 ± 0.124**^**A**^	**3.830 ± 0.251**^**B**^	**< 0.001**
n-9 PUFA	0.416 ± 0.023	0.492 ± 0.096	0.473 ± 0.028	0.396 ± 0.020	0.377
DMA	0.128 ± 0.013	0.287 ± 0.109	0.170 ± 0.049	0.192 ± 0.027	0.133
**UFA/SFA**	**1.567 ± 0.050**^**A**^	**1.737 ± 0.032**^**B**^	**1.627 ± 0.043**^**AB**^	**1.707 ± 0.037**^**AB**^	**0.045**
**n-3/n-6 PUFA**	**0.447 ± 0.017**^**AB**^	**0.517 ± 0.021**^**C**^	**0.437 ± 0.014**^**A**^	**0.495 ± 0.021**^**BC**^	**0.006**
**Prod/prec n-6**	**2.304 ± 0.148**^**A**^	**3.403 ± 0.398**^**B**^	**2.231 ± 0.160**^**A**^	**3.683 ± 0.357**^**B**^	**0.001**
**Prod/prec n-3**	**16.628 ± 4.026**^**B**^	**16.064 ± 1.271**^**A**^	**15.819 ± 2.967**^**AB**^	**15.089 ± 1.153**^**A**^	**0.045**

Results are presented as mean ± SEM. Individual components are listed in the order of ascending chromatographic retention time. Alkenyl chain is detected as DMA. *OA* Orotic acid, *LPS* Lipopolysaccharide, *DMA* Plasmalogen alkenyl chain-derived dimethyl acetal derivative, *i* = iso, *ai* = anteiso, *c* = cis, *t* = trans, *SFA* Saturated fatty acid, *MUFA* Monounsaturated fatty acid, *PUFA* Polyunsaturated fatty acid, UFA = MUFA + PUFA, prod = product, prec = precursor, means with different superscript letters are significantly different from each other (Kruskal–Wallis ANOVA). Fatty acids with significant differences between means are shown in bold

The culture media of OA-, LPS- or OA–LPS-treated HepG2 cells did not differ from control medium in their FA profiles (Table 3). This was also clearly visualized by the discriminant analysis including all media (Fig. 4c). Compared to raw medium, the other media had higher percentages of 17:0, 20:0, 24:0, several C14–18 monounsaturated FA (MUFA), 24:1n-9 and most C20–22 PUFA, such as 20:3n-6, 20:4n-6, 20:5n-3 and 22:6n-3 (Table 3). Furthermore, their n-3 and n-6 PUFA sums, UFA/SFA ratios and product/precursor ratios of n-3 and n-6 PUFA were higher. In contrast, the media of OA-, LPS- and OA–LPS-treated cells had lower proportions of, e.g., DMA 18:0, FA 18:0, 18:1n-9 and 20:4n-3, and also total SFA compared to raw medium. The DMA sum was lower in the media of OA–LPS-treated cells compared to raw medium, and DMA 18:1n-9 was lower in the media of control, LPS- and OA–LPS-treated cells. When raw medium was excluded from the discriminant analysis, the media of control and OA–LPS-treated cells were visibly apart from each other and from the media of OA- and LPS-treated cells that were grouped together (Fig. 4d). The individual FA most strongly separating the treatments were 22:5n-3, 20:4n-6, 22:6n-3, 18:2n-7 and 16:1n-7. Overall, the analysis classified 100% of the samples correctly into their respective treatments.

**Table 3:** Fatty acyl and alkenyl chain profiles (mol-%) in raw medium and growth media of HepG2 cells

	Raw medium	Control medium	OA medium	LPS medium	OA–LPS medium	*p*
14:0	2.815 ± 0.290	2.979 ± 0.118	2.913 ± 0.198	2.830 ± 0.110	2.734 ± 0.109	0.539
**14:1n-5**	**0.266 ± 0.052**^**A**^	**1.048 ± 0.162**^**B**^	**1.499 ± 0.387**^**B**^	**1.001 ± 0.138**^**B**^	**0.937 ± 0.118**^**B**^	**0.002**
15:0	1.139 ± 0.200	0.876 ± 0.059	0.992 ± 0.102	0.805 ± 0.044	0.866 ± 0.041	0.417
DMA 16:0	0.228 ± 0.023	0.314 ± 0.080	0.508 ± 0.142	0.291 ± 0.022	0.227 ± 0.036	0.131
16:0	29.947 ± 0.775	28.759 ± 0.505	27.390 ± 0.640	28.773 ± 0.494	28.086 ± 0.373	0.147
**16:1n-9**	**0.559 ± 0.048**^**A**^	**2.002 ± 0.105**^**B**^	**1.924 ± 0.059**^**B**^	**1.935 ± 0.081**^**B**^	**1.841 ± 0.089**^**B**^	**0.002**
**16:1n-7**	**1.803 ± 0.211**^**A**^	**5.009 ± 0.192**^**B**^	**4.854 ± 0.357**^**B**^	**4.828 ± 0.107**^**B**^	**4.492 ± 0.218**^**B**^	**0.001**
16:1n-5	0.665 ± 0.188	0.611 ± 0.040	0.754 ± 0.104	0.638 ± 0.041	0.567 ± 0.048	0.629
**17:0*i***	**1.039 ± 0.133**^**B**^	**0.316 ± 0.047**^**A**^	**0.529 ± 0.108**^**A**^	**0.329 ± 0.044**^**A**^	**0.334 ± 0.045**^**A**^	**0.002**
**17:0*ai***	**0.512 ± 0.060**^**A**^	**0.649 ± 0.049**^**AB**^	**0.804 ± 0.066**^**B**^	**0.657 ± 0.062**^**B**^	**0.639 ± 0.042**^**B**^	**0.032**
**17:0**	**0.453 ± 0.113**^**A**^	**1.120 ± 0.155**^**B**^	**1.046 ± 0.171**^**B**^	**0.961 ± 0.133**^**B**^	**0.865 ± 0.059**^**B**^	**0.027**
**17:1n-8**	**0.271 ± 0.087**^**A**^	**0.892 ± 0.043**^**B**^	**0.985 ± 0.069**^**B**^	**0.920 ± 0.050**^**B**^	**0.952 ± 0.056**^**B**^	**0.003**
**DMA 18:0**	**0.537 ± 0.103**^**B**^	**0.295 ± 0.032**^**A**^	**0.321 ± 0.068**^**A**^	**0.262 ± 0.032**^**A**^	**0.244 ± 0.020**^**A**^	**0.031**
**DMA 18:1n-9**	**0.291 ± 0.045**^**B**^	**0.128 ± 0.017**^**A**^	**0.184 ± 0.040**^**AB**^	**0.118 ± 0.020**^**A**^	**0.112 ± 0.012**^**A**^	**0.009**
DMA 18:1n-7	0.096 ± 0.019	0.085 ± 0.016	0.114 ± 0.021	0.066 ± 0.007	0.083 ± 0.009	0.428
**18:0**	**19.532 ± 0.451**^**B**^	**11.025 ± 0.555**^**A**^	**11.063 ± 0.691**^**A**^	**11.645 ± 0.378**^**A**^	**12.520 ± 0.689**^**A**^	**0.001**
**18:1n-9**	**24.307 ± 0.871**^**B**^	**17.579 ± 0.768**^**A**^	**17.647 ± 0.628**^**A**^	**18.088 ± 0.577**^**A**^	**18.160 ± 0.554**^**A**^	**0.004**
**18:1n-7**	**4.110 ± 0.210**^**A**^	**7.426 ± 0.414**^**B**^	**6.466 ± 0.262**^**B**^	**7.181 ± 0.283**^**B**^	**6.865 ± 0.437**^**B**^	**0.001**
**18:1n-5**	**1.376 ± 0.159**^**B**^	**0.643 ± 0.043**^**A**^	**0.647 ± 0.063**^**A**^	**0.561 ± 0.026**^**A**^	**0.623 ± 0.032**^**A**^	**0.002**
18:2n-7	0.447 ± 0.042	0.540 ± 0.062	0.537 ± 0.062	0.511 ± 0.021	0.426 ± 0.060	0.604
18:2n-6	1.992 ± 0.129	2.600 ± 0.232	2.882 ± 0.229	2.617 ± 0.173	2.461 ± 0.324	0.167
18:3n-6	0.717 ± 0.136	0.387 ± 0.088	0.540 ± 0.101	0.370 ± 0.077	0.330 ± 0.052	0.098
19:1n-8	0.176 ± 0.034	0.117 ± 0.015	0.111 ± 0.015	0.096 ± 0.010	0.092 ± 0.012	0.105
18:3n-3	0.427 ± 0.063	0.345 ± 0.032	0.399 ± 0.059	0.304 ± 0.017	0.292 ± 0.026	0.284
18:2*c*9*t*11	0.138 ± 0.018	0.203 ± 0.032	0.242 ± 0.055	0.216 ± 0.059	0.237 ± 0.052	0.773
**20:0**	**0.193 ± 0.042**^**A**^	**0.448 ± 0.032**^**B**^	**0.458 ± 0.045**^**B**^	**0.440 ± 0.042**^**B**^	**0.436 ± 0.027**^**B**^	**0.009**
20:1n-11	0.106 ± 0.026	0.224 ± 0.056	0.301 ± 0.050	0.257 ± 0.055	0.214 ± 0.051	0.214
20:1n-9	0.301 ± 0.049	0.483 ± 0.059	0.503 ± 0.113	0.441 ± 0.024	0.422 ± 0.021	0.093
20:1n-7	0.187 ± 0.036	0.318 ± 0.043	0.319 ± 0.051	0.301 ± 0.038	0.301 ± 0.032	0.293
20:2n-9	0.173 ± 0.032	0.222 ± 0.036	0.287 ± 0.080	0.185 ± 0.016	0.147 ± 0.020	0.465
**20:2n-6**	**0.369 ± 0.052**^**A**^	**0.657 ± 0.089**^**B**^	**0.619 ± 0.052**^**B**^	**0.674 ± 0.057**^**B**^	**0.630 ± 0.027**^**B**^	**0.029**
20:3n-9	0.208 ± 0.052	0.156 ± 0.020	0.201 ± 0.027	0.188 ± 0.024	0.190 ± 0.027	0.758
**20:3n-6**	**0.539 ± 0.045**^**A**^	**1.339 ± 0.041**^**B**^	**1.459 ± 0.054**^**B**^	**1.276 ± 0.055**^**B**^	**1.450 ± 0.047**^**B**^	**< 0.001**
**20:4n-6**	**0.999 ± 0.092**^**A**^	**2.912 ± 0.124**^**BC**^	**3.027 ± 0.156**^**BC**^	**2.920 ± 0.092**^**B**^	**3.290 ± 0.085**^**C**^	**< 0.001**
**20:4n-3**	**0.960 ± 0.117**^**B**^	**0.246 ± 0.035**^**A**^	**0.237 ± 0.020**^**A**^	**0.212 ± 0.014**^**A**^	**0.210 ± 0.018**^**A**^	**0.002**
**20:5n-3**	**0.299 ± 0.059**^**A**^	**0.526 ± 0.059**^**B**^	**0.598 ± 0.055**^**B**^	**0.532 ± 0.031**^**B**^	**0.547 ± 0.046**^**B**^	**0.043**
22:0	0.580 ± 0.094	0.599 ± 0.063	0.554 ± 0.036	0.534 ± 0.045	0.585 ± 0.063	0.908
22:1n-11	0.144 ± 0.017	0.312 ± 0.044	0.272 ± 0.036	0.256 ± 0.037	0.337 ± 0.114	0.111
22:1n-9	0.028 ± 0.008	0.064 ± 0.010	0.055 ± 0.008	0.054 ± 0.006	0.049 ± 0.007	0.095
22:1n-7	0.106 ± 0.012	0.173 ± 0.043	0.139 ± 0.028	0.134 ± 0.014	0.120 ± 0.013	0.681
22:4n-6	0.074 ± 0.011	0.183 ± 0.057	0.233 ± 0.058	0.268 ± 0.117	0.196 ± 0.047	0.052
**22:5n-3**	**0.169 ± 0.034**^**A**^	**1.387 ± 0.056**^**B**^	**1.542 ± 0.064**^**BC**^	**1.497 ± 0.091**^**BC**^	**1.686 ± 0.083**^**C**^	**< 0.001**
**24:0**	**0.237 ± 0.048**^**A**^	**0.764 ± 0.260**^**B**^	**0.600 ± 0.047**^**B**^	**0.537 ± 0.051**^**B**^	**0.575 ± 0.066**^**B**^	**0.012**
**22:6n-3**	**0.387 ± 0.054**^**A**^	**2.000 ± 0.157**^**B**^	**2.032 ± 0.097**^**B**^	**2.079 ± 0.131**^**B**^	**2.326 ± 0.085**^**B**^	**0.001**
**24:1n-9**	**0.097 ± 0.014**^**A**^	**1.040 ± 0.205**^**B**^	**1.215 ± 0.136**^**B**^	**1.214 ± 0.141**^**B**^	**1.305 ± 0.131**^**B**^	**0.001**
**SFA**	**56.448 ± 0.435**^**B**^	**47.535 ± 1.294**^**A**^	**46.349 ± 1.032**^**A**^	**47.510 ± 0.826**^**A**^	**47.640 ± 0.852**^**A**^	**0.005**
MUFA	34.504 ± 0.541	37.941 ± 1.321	37.690 ± 0.966	37.905 ± 0.857	37.278 ± 1.123	0.176
**PUFA**	**7.898 ± 0.368**^**A**^	**13.704 ± 0.344**^**B**^	**14.835 ± 0.372**^**B**^	**13.848 ± 0.352**^**B**^	**14.416 ± 0.583**^**B**^	**< 0.001**
**n-6 PUFA**	**4.690 ± 0.153**^**A**^	**8.079 ± 0.309**^**B**^	**8.760 ± 0.396**^**B**^	**8.124 ± 0.303**^**B**^	**8.356 ± 0.434**^**B**^	**0.001**
**n-3 PUFA**	**2.242 ± 0.220**^**A**^	**4.504 ± 0.199**^**B**^	**4.807 ± 0.147**^**B**^	**4.624 ± 0.248**^**B**^	**5.060 ± 0.200**^**B**^	**0.001**
n-9 PUFA	0.381 ± 0.082	0.378 ± 0.046	0.488 ± 0.104	0.373 ± 0.023	0.337 ± 0.044	0.713
**DMA**	**1.151 ± 0.173**^**B**^	**0.821 ± 0.112**^**B**^	**1.127 ± 0.255**^**B**^	**0.737 ± 0.062**^**AB**^	**0.666 ± 0.047**^**A**^	**0.041**
**UFA/SFA**	**0.752 ± 0.015**^**A**^	**1.100 ± 0.051**^**B**^	**1.144 ± 0.046**^**B**^	**1.096 ± 0.036**^**B**^	**1.093 ± 0.039**^**B**^	**0.005**
n-3/n-6 PUFA	0.480 ± 0.049	0.567 ± 0.038	0.563 ± 0.033	0.580 ± 0.039	0.624 ± 0.037	0.324
**Prod/prec n-6**	**0.502 ± 0.030**^**A**^	**1.198 ± 0.095**^**B**^	**1.090 ± 0.060**^**B**^	**1.160 ± 0.068**^**B**^	**1.907 ± 0.461**^**B**^	**0.001**
**Prod/prec n-3**	**1.736 ± 0.188**^**A**^	**7.878 ± 0.708**^**B**^	**7.811 ± 0.840**^**B**^	**8.778 ± 0.566**^**BC**^	**10.334 ± 0.597**^**C**^	**< 0.001**

Results are presented as mean ± SEM. Individual components are listed in the order of ascending chromatographic retention time. Alkenyl chain is detected as DMA. *OA* Orotic acid, *LPS* Lipopolysaccharide, *DMA* Plasmalogen alkenyl chain-derived dimethyl acetal derivative, *i* = iso, *ai* = anteiso, *c* = cis, *t* = trans, *SFA* Saturated fatty acid, *MUFA* Monounsaturated fatty acid, *PUFA* Polyunsaturated fatty acid, UFA = MUFA + PUFA, prod = product, prec = precursor, means with different superscript letters are significantly different from each other (Kruskal–Wallis ANOVA). Fatty acids with significant differences between treatments and raw medium are shown in bold

## Discussion

Several previous studies have revealed that OA fails to induce fatty liver in species other than rats [17, 18], in which the effect has been clearly established since 1950’s [24]. Nonetheless, Jung et al. [21] reported similarities in OA-induced effects on lipogenic transcription factors between human HCC and rat primary hepatocytes, and the authors speculated that OA may be capable of causing fatty liver also in humans. However, in our experiments OA did not cause evident intracellular lipid accumulation in HepG2 cells as revealed by fluorescent Nile red staining and TAG measurement. We also studied the mRNA expression levels of ACACA, ACACB and FASN to see whether OA affects DNL, which has previously been the case in SK-Hep1 HCC cells [21]. Interestingly, although actual lipidosis was not observed, OA did have an increasing effect on the mRNA expression of acetyl-CoA carboxylase, which converts acetyl CoA to malonyl CoA as the irreversible committed step of FA synthesis leading to DNL. Despite this, OA did not cause any statistically significant increase in FASN mRNA levels or enzyme activities, while the expression levels of IL-6 increased drastically. Furthermore, the results of the Human Fatty Liver Array did not show DNL-related differences between control and OA-treated cells. Altogether, our findings indicate that OA triggers an inflammatory response and potentially promotes the first stage of DNL but does not cause definite lipidosis in HepG2 cells.

LPS did not further aggravate the phenomena related to lipid accumulation or inflammation triggered by OA, although elevated circulating LPS levels and increased hepatic expression of Toll-like receptor 4 (TLR4) have previously been observed [25–28]. Even though LPS-induced steatosis is characterized by hepatic insulin resistance and the higher expression of tumor necrosis factor-*α*, IL-1 and IL-6 [29, 30], similar to the high-fat diet model, there are also some features that distinguish it from common NAFLD models. The LPS-induced lipid accumulation mainly occurs in the hepatocytes around the portal vein, whereas high-fat diet induces lipidosis around the central vein [30]. In addition, the expression of SREBP-1c and PPAR-*γ*, the main transcription factors regulating the activity of lipogenic enzymes, is inhibited, indicating that LPS-induced liver steatosis is not characterized by increased DNL. In our experiments, LPS alone did not affect the levels of ACACA, ACACB nor FASN in HepG2 cells, indicating that it does not induce DNL-mediated steatosis in HCC cell model either. The synergic effects of LPS and diets inducing fatty liver have been addressed in a few in vivo studies. In rats on high-disaccharide diet, LPS administration significantly aggravated fat accumulation and increased hepatic DNL, while in LDL receptor-deficient mice the combination of LPS and high 16:0 and fat diet promoted hepatic inflammation [31, 32]. OA, LPS or their combination did not induce definite lipidosis in the present study, indicating that the HepG2 model does not extend to the whole cascade of lipid accumulation but could still be useful to examine the first stages of the disease.

As we were able to demonstrate that OA failed to cause lipid accumulation, it became possible to concentrate on phenomena protecting the cells from NAFLD-like pathology. To study potential mechanisms involved in the prevention of OA-mediated lipidosis, we analyzed the detailed FA composition of cells and their culture media. In OA-treated cells, the proportions of 16:0 decreased and those of 16:1n-7 remained relatively high, which suggests stimulated Δ9-desaturation. This change was likely mediated by SCD1, the enzyme catalyzing the formation of 16:1n-7 and 18:1n-9 from their SFA precursors 16:0 and 18:0, respectively [33]. The substrates available from medium and the cell-specific relative affinities of the ER desaturase and elongase enzymes for the de novo-synthesized substrate 16:0 determine whether the main MUFA produced is 16:1n-7 (efficient Δ9-desaturation) or 18:1n-9 (efficient elongation to 18:0 followed by Δ9-desaturation) [34]. In hepatocytes, 16:1n-7 reduces lipogenesis and improves insulin sensitivity preventing lipid accumulation, while 18:1n-9 promotes lipogenesis and development of steatosis and increases glucose intolerance [35]. Thus, the enhanced conversion of 16:0 to 16:1n-7 in OA-treated HepG2 cells and the consequent signaling leading to reduced lipogenesis may be central factors preventing steatosis and other types of dysfunction in this model. Highly active Δ9-desaturation has previously been reported both in surgically resected HCC and HCC cell lines, including HepG2, and it has been suggested to act as a mechanism alleviating chemotherapy-induced apoptosis [36]. Accordingly, 16:1n-7 has been shown to reduce 16:0-induced apoptosis and inflammation in human and mouse hepatocytes [37, 38], and to reduce hepatic steatosis in mice in vivo [39]. Thus, our findings lead to a plausible hypothesis that via active Δ9-desaturation, the OA- and OA–LPS-treated HepG2 cells could resist the accumulation of lipids and, at the same time, help to decrease the levels of toxic SFA. Thus, active SCD1 function would alleviate ER stress and attenuate unfolded protein response. Simultaneously, the decreased SFA levels would mitigate TLR-mediated pro-inflammatory responses [35, 40]. These pathways link the SCD1 function with a wide spectrum of responses regulating metabolism, cell stress and inflammation.

The roles of SCD1 and its product 16:1n-7 have been studied in patients with NASH and NAFLD. In NASH, increased SCD1 activity, measured by the increased 16:1n-7/16:0 ratio in plasma, and increased hepatic SCD1 expression were reported [41, 42]. Patients with NAFLD also had increased circulating levels of 16:1n-7 [43]. However, the hepatic levels of 16:0 previously increased in NASH patients as they did in high-fat diet-fed mice [41, 44]. In hepatocytes, Δ9-desaturation may also be enhanced as a response to increased DNL, continuously providing the substrate 16:0 [45]. However, in our HepG2 experiments, although the 16:0 proportions in OA-treated cells decreased and SCD1 expression was enhanced, DNL was not elevated. These results suggest that in the absence of stimulated DNL, the conversion of 16:0 to 16:1n-7 via SCD1 could be effective enough to reduce lipogenesis and to reverse the accumulation of toxic 16:0 in OA-treated cells. The Δ9-desaturation cascade could thus provide a promising research target for preventing lipotoxicity and the consequent fatty liver disease.

The divergent FA profiles of the differently treated cells were not directly mirrored by the FA profiles of the culture media. The media supplemented with 10% fetal bovine serum commonly has an order of magnitude higher FA contents than cultured mammalian cells [46], and the media were replaced during the 5-day incubations. Thus, provided that the differences in the FA profiles of the media reflected differences in the cellular FA metabolism, milder differences of FA composition were expected to occur between the media than the differently treated cells. In addition, FA are recycled between the cells and media. Secreted FA are taken up by the cells in selective processes either utilizing the free FA or FA incorporated into lipoproteins, then hydrolyzing the lipids and releasing free FA by hepatic lipases, and finally transporting the free FA into the cells by the aid of several FA-binding and transport proteins [47, 48]. Both FA hydrolysis and their protein-mediated uptake have preferences for FA structure and are known to prefer 18:1n-9 [49]. Phospholipid turnover also favors C18 FA for the sn-1 position of the molecules [50]. Due to this selectivity, the medium FA profiles do not necessarily match the cellular profiles and the fluxes may not lead to equilibrium. For example, HepG2 cells clearly transferred 16:1n-7 to the medium but 18:1n-9, apparently due to its efficient recycling, reached similar percentages in the cells and the culture media.

The proportions of 18:0, 18:1n-9 and total SFA decreased in culture media compared to raw medium, suggesting the active uptake of these FA by HepG2 cells. Despite this, the SFA percentages decreased in OA- and OA–LPS-treated cells. In macrophages, the SFA-induced inflammatory response can be inhibited by the pre-treatment with PUFA and 18:1n-9 [51]. Furthermore, in hepatocytes, the presence of 18:1n-9 greatly reduces 16:0-mediated lipotoxicity [52]. Thus, the selective incorporation of 18:1n-9 to HepG2 cells could have alleviated inflammatory signaling. Despite the decreased 18:0 levels in culture media and OA-induced Δ9-desaturation activity, the intracellular 18:1n-9 proportions did not increase by OA exposure. One option is that incorporated 18:0 was not directed to 18:1n-9 synthesis, although active 16:1n-7 synthesis from 16:0 was supported. Thus, lipogenesis due to elevated 18:1n-9 [35] was not realized. Second, the efficient recycling of 18:1n-9 between the cellular and medium FA pools may have limited the rise of 18:1n-9 inside the cells, as discussed previously. Third, 18:1n-9 is used as the precursor for the longest MUFA (that have their double bond in the n-9 position, and rarely in the n-7 position that would be the case if 16:1n-7 had been the precursor) incorporated to sphingolipids, which also limits the accumulation of 18:1n-9 in the cells. Indeed, although the proportions of 20:1n-9 remained unchanged, the percentages of 22:1n-9 and 24:1n-9 slightly increased in OA- and/or OA–LPS-treated cells compared to control and/or LPS-treated cells. These findings indicate that OA exposure might have slightly increased the activities of elongation of very-long-chain FA (ELOVL) 1 and 3, which promote the synthesis of 20:1n-9, 22:1n-9 and 24:1n-9, the downstream products of 18:1n-9 that are utilized especially as sphingolipid acyl chains in HepG2 and other hepatic cells [53]. Thus, we can hypothesize that ELOVL1 and 3 may take part in maintaining 18:1n-9 levels unchanged in cells exposed to OA, and the possible connections to sphingolipid metabolism of the cells recall further studies.

Various FA, including n-6 and n-3 PUFA, 16:1n-9, 16:1n-7, 18:1n-7, 20:4n-6, 20:5n-3 and 22:6n-3 were probably secreted from HepG2 cells into the medium. In addition, the proportions of several long-chain PUFA, such as 20:4n-6, 20:5n-3 and 22:6n-3 increased in both OA- and OA–LPS-treated cells. Although NAFLD has been reported to be accompanied by reduced levels of long-chain PUFA and higher n-6/n-3 PUFA ratios in the liver [10], OA exposure increased the n-3 PUFA percentages in HepG2 cells, indicating a higher production of these PUFA, which mediate anti-inflammatory actions [12, 15, 54]. Together, this finding and the increased IL-6 expression suggest that in addition to inflammatory cascades in the OA-treated hepatocytes, protective biochemical phenomena may also arise. HepG2 cells have a good capacity to produce these highly unsaturated long-chain PUFA from their C18 PUFA precursors [55]. When incorporated into cell membranes, PUFA can alter membrane fluidity and distribution of phospholipid molecular species between the raft and non-raft fractions [56], eventually altering the microenvironment and activity of membrane-associated receptors and enzymes [57]. The long-chain PUFA 22:6n-3 has been associated with diminished recruitment of TLR4 into membrane raft fractions and subsequently attenuated pro-inflammatory response to LPS or 12:0 [58]. Furthermore, as PUFA diminish the expression of SREBP-1c [59], increased PUFA levels may also account for inhibited lipogenic transformation in OA- and OA–LPS-treated cells. Elevated 22:6n-3, requiring peroxisomal partial *β*-oxidation of 24:6n-3 as the last step of its synthesis [60], suggests that OA may promote peroxisomal PUFA metabolism. Increased proportions of long-chain PUFA could also be related to accelerated synthesis of membrane phospholipids triggered by ER stress [61], a characteristic of NAFLD [62]. Moreover, n-6 and n-3 PUFA are precursors to short-lived pro-inflammatory and resolving lipid mediators that could play roles in liver inflammation [63]. While the total proportions of PUFA increased in OA- and OA–LPS-treated cells, the total SFA decreased and total MUFA remained unaffected. Thus, the ratio of MUFA to SFA was elevated, consistent with the increased SCD1 expression level. The total MUFA may have been limited by increased levels of PUFA, which are known to inhibit MUFA synthesis and to reduce DNL [64].

The percentages of 20:4n-6 increased in OA- and OA–LPS-treated cells, as well as in their culture media. Increased 20:4n-6 levels have been documented in the liver of rats fed methionine choline-deficient diet [65], unlike previous reports on reduced or unchanged levels in human NASH [10, 43]. The only other FA elevated along the n-6 PUFA pathway was 20:3n-6. Given that the modest increase in 22:4n-6 proportions in OA-treated cells did not reach statistical significance, it is plausible that 20:4n-6 would not have been actively utilized for the production of 22:4n-6. In HepG2 cells, 20:4n-6 reduces the expression of SREBP-1 and FASN [66]. Furthermore, 20:4n-6 has been associated with the synthesis and secretion of lipoproteins from PUFA-enriched ER membranes in hepatocytes [67, 68]. Thus, the increased 20:4n-6 and total PUFA levels in HepG2 suggest that OA-treated cells could alleviate lipidosis by increasing the lipid secretion as nascent lipoprotein-like particles from ER. Moreover, OA exposure caused an increase in DMA 18:0 (derived from plasmalogens), and FA 20:5n-3 and 22:6n-3 proportions. For instance, plasmalogen-deficient cells are characterized by alterations in plasma membrane and dilatation of ER and Golgi compartments [69], which emphasizes the crucial role of these membrane components in membrane fusion, vesicle formation and trafficking. Particularly, several lines of evidence have prompted the hypothesis that plasmalogens also promote the release of extracellular vesicles [70]. Furthermore, 22:6n-3 treatment of differentiated mouse 3T3-L1 adipocytes has been suggested to increase the release of adiponectin-containing extracellular vesicles [71]. Collectively, the observed increases in DMA 18:0 and PUFA 22:6n-3 proportions suggest that, in addition to promoted lipoprotein secretion, OA may trigger enhanced production of extracellular vesicles from HepG2. Interestingly, 16:0, present in high levels in the circulation of NASH patients [72], induces the large-quantity production and release of extracellular vesicles that can play a role in several key events in NAFLD pathogenesis [73]. Indeed, NAFLD patients have been documented to have elevated levels of circulating extracellular vesicles [74]. Based on these findings, it is tempting to speculate that the release of extracellular vesicles may be one pathway mitigating the effects of potentially toxic FA, also upon OA exposure. The roles of lipoproteins and extracellular vesicles in the prevention of OA-mediated lipidosis should be evaluated by further research.

## Conclusions

Our data indicate that although OA possibly promotes the first stage of DNL, it fails to cause a definite lipogenic transformation in HepG2 cells. Modifications of FA metabolism may be involved in this prevention of OA-mediated lipidosis. Reduced proportions of 16:0, together with increased SCD1 mRNA expression and relatively high proportions of 16:1n-7 suggest that active Δ9-desaturation may be a mechanism limiting lipogenesis, ER stress and the accumulation of toxic 16:0. Inflammatory signaling may be reduced by the increased production of long-chain n-3 PUFA and the active incorporation of certain FA, including 18:1n-9, into cells. In addition, increased proportions of long-chain PUFA and DMA 18:0 suggest that OA exposure may cause increased secretion of lipoproteins and extracellular vesicles, which could provide promising targets of translational research for preventing fatty liver disease.

## Supplementary information

**Additional file 1 **Human fatty liver PCR array results. RT^2^ Profiler™ PCR Array analysis showing the upregulated genes in OA-treated cells. The mean fold changes of three independent experiments are shown. *p = 0.046.

## Data Availability

The datasets used and/or analyzed during the current study are available from the corresponding author on reasonable request.

## Abbreviations

ACACA: Acetyl-coenzyme A carboxylase *α*
ACACB: Acetyl-coenzyme A carboxylase *β*
acetyl-CoA: acetyl-coenzyme A
AMPK: AMP-activated protein kinase
ANOVA: Analysis of variance
DMA: Dimethyl acetal derivative of alkenyl chain
DNL: De novo lipogenesis
ELOVL: Elongation of very-long-chain fatty acid
ER: Endoplasmic reticulum
FA: Fatty acid
FAME: Fatty acid methyl ester
FASN: Fatty acid synthase
FID: Flame ionization detector
GC: Gas chromatography
HCC: Hepatocellular carcinoma
IL: Interleukin
LDL: Low-density lipoprotein
LPS: Lipopolysaccharide
MSD: Mass selective detector
mTOR: mammalian target of rapamycin
MUFA: Monounsaturated fatty acid
NAFLD: Non-alcoholic fatty liver disease
NASH: Non-alcoholic steatohepatitis
OA: Orotic acid
PBS: Phosphate buffered saline
PI: Propidium iodide
PPAR-*α*: Peroxisome proliferator-activated receptor-*α*
PUFA: Polyunsaturated fatty acid
SCD1: Stearoyl-Coenzyme A desaturase 1
SFA: Saturated fatty acid
SREBP-1c: Sterol regulatory element binding protein-1c
TAG: Triacylglycerol
TLR4: Toll-like receptor 4
UFA: Unsaturated fatty acid
VLDL: Very-low-density lipoprotein
