# Supplementary material for: Orotic acid-treated hepatocellular carcinoma cells resist steatosis by modification of fatty acid metabolism
Source: Lipids Health Dis. 2020 Apr 13;19:70. doi: 10.1186/s12944-020-01243-5 (PMC7155272; doi:10.1186/s12944-020-01243-5)
Supplement: Supplementary file 1 — Additional file 1 Human fatty liver PCR array results. RT2 Profiler™ PCR Array analysis showing the upregulated genes in OA-treated cells. The mean fold changes of three independent experiments are shown. *p = 0.046. [file 12944_2020_1243_MOESM1_ESM.docx]

**Additional file 1**

**
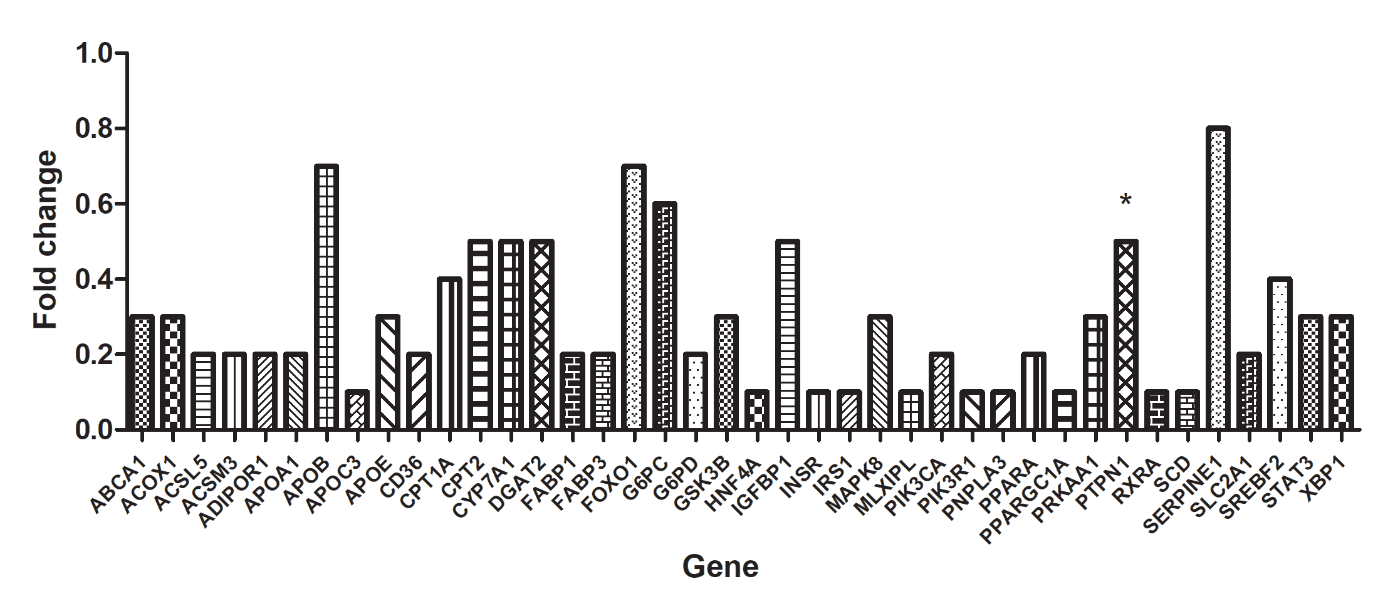
**

**Genes**

ABCA1, ATP-binding cassette, sub-family A (ABC1), member 1

ACOX1, Acyl-CoA oxidase 1, palmitoyl

ACSL5, Acyl-CoA synthetase long-chain family member 5

ACSM3, Acyl-CoA synthetase medium-chain family member 3

ADIPOR1, Adiponectin receptor 1

APOA1, Apolipoprotein A-I

APOB, Apolipoprotein B (including Ag(x) antigen)

APOC3, Apolipoprotein C-III

APOE, Apolipoprotein E

CD36, CD36 molecule (thrombospondin receptor)

CPT1A, Carnitine palmitoyltransferase 1A (liver)

CPT2, Carnitine palmitoyltransferase 2

CYP7A1, Cytochrome P450, family 7, subfamily A, polypeptide 1

DGAT2, Diacylglycerol O-acyltransferase 2

FABP1, Fatty acid binding protein 1, liver

FABP3, Fatty acid binding protein 3, muscle and heart (mammary-derived growth

inhibitor)

FOXO1, Forkhead box O1

G6PC, Glucose-6-phosphatase, catalytic subunit

G6PD, Glucose-6-phosphate dehydrogenase

GSK3B, Glycogen synthase kinase 3 beta

HNF4A, Hepatocyte nuclear factor 4, alpha

IGFBP1, Insulin-like growth factor binding protein 1

INSR, Insulin receptor

IRS1, Insulin receptor substrate 1

MAPK8, Mitogen-activated protein kinase 8

MLXIPL, MLX interacting protein-like

PIK3CA, Phosphoinositide-3-kinase, catalytic, alpha polypeptide

PIK3R1, Phosphoinositide-3-kinase, regulatory subunit 1 (alpha)

PNPLA3, Patatin-like phospholipase domain containing 3

PPARA, Peroxisome proliferator-activated receptor alpha

PPARGC1A, Peroxisome proliferator-activated receptor gamma, coactivator 1 alpha

PRKAA1, Protein kinase, AMP-activated, alpha 1 catalytic subunit

PTPN1, Protein tyrosine phosphatase, non-receptor type 1

RXRA, Retinoid X receptor, alpha

SCD, Arylacetamide deacetylase

SERPINE1, Serpin peptidase inhibitor, clade E (nexin, plasminogen activator inhibitor type

1), member 1

SLC2A1, Solute carrier family 2 (facilitated glucose transporter), member 1

SREBF2, Sterol regulatory element binding transcription factor 2

STAT3, Signal transducer and activator of transcription 3 (acute-phase response factor)

XBP1, X-box binding protein 1
